# Biopsychosocial care and the physiotherapy encounter: physiotherapists’ accounts of back pain consultations

**DOI:** 10.1186/1471-2474-14-65

**Published:** 2013-02-19

**Authors:** Tom Sanders, Nadine E Foster, Annette Bishop, Bie Nio Ong

**Affiliations:** 1 Arthritis Research UK Primary Care Centre, Primary Care Sciences, Keele University, Staffordshire, ST5 5BG, UK

**Keywords:** Physiotherapy, Back pain, Biopsychosocial, Clinician-patient communication

## Abstract

**Background:**

The physiotherapy profession has undergone a paradigmatic shift in recent years, where a ‘biopsychosocial’ model of care has acquired popularity in response to mounting research evidence indicating better patient outcomes when used alongside traditional physiotherapy. However, research has not examined how this new dimension to traditional physical therapy is implemented within the therapeutic consultation.

**Methods:**

The study aimed to investigate physiotherapists’ reported approaches to back pain care in the context of increasing pressure to address patients’ psychosocial concerns. A secondary analysis of semi-structured qualitative interviews with 12 UK physiotherapists was conducted. Respondents were sampled from a national survey, to include a broad mix of physiotherapists. Data were analysed thematically, adopting the constant comparative methodology.

**Results:**

The combination of traditional physical therapy with a broader biopsychosocial approach presented significant challenges. Physiotherapists responded by attempting to navigate patients’ biopsychosocial problems through use of various strategies, such as setting boundaries around their clinical role and addressing lay health beliefs of patients through the provision of reassurance and lifestyle advice.

**Conclusions:**

As psychosocial issues, alongside biomechanical factors, command a prominent place within the back pain consultation, physiotherapists may benefit from further specific training and mentoring support in identifying specific strategies for combining the best of traditional physiotherapy approaches with greater focus on patients’ beliefs, fears and social context.

## Background

The National Institute for Health and Clinical Excellence [1] has called for greater use of the biopsychosocial model to manage patients with low back pain (LBP) alongside early detection and treatment of symptoms. However, evidence that the advice is adhered to in clinical practice is scarce. The question that we sought to address in this secondary analysis was how do therapists attempt to resolve the competing pressure to address both the mechanical and the psychosocial components of back pain care with the patient. Though we recognise that the separation between these two components is somewhat artificial, and non-specific low back pain is multidimensional. The biopsychosocial model acknowledges the patient as a whole, their social, cultural and environmental context that shapes an individual’s response to illness, in essence a patient-centred healthcare system. There is a wide misconception that being patient centred means sharing all information and all decisions. Patient-centeredness may instead involve taking into account the patient’s desire for information and for sharing decision making and responding appropriately. Thus, any discussion about therapeutic decisions will begin with the patient’s perspective [2]. Evidence from recent studies indicates that physiotherapy has begun to embrace the biopsychosocial model, though this still raises the question of how this new approach has been utilised in the management of back pain [3].

More recently, the term patient-centred care has been used in policy documents such as The Health and Social Care Bill 2011 in the UK [4], and is often linked to notions of choice and empowerment. Recent studies, however, suggest that physiotherapists may still be poorly prepared to address the challenges presented by patients with low back pain, including the expectation that they provide a more patient focused and broader biopsychosocial approach to care [5-7]. For example, elements of a biopsychosocial approach that appear to be lacking, in physiotherapy practice, as reported in recent research are: a) explanation of a clear diagnosis or cause for the symptoms to patients [5-7] and b) different expectations about the benefits of physical therapy, beliefs about the natural course of back pain, and the degree of responsibility patients should exercise over their care [2,8,9]. However, the most effective means of delivering a biopsychosocial approach is not well understood [10,11].

Within physiotherapy an increasing body of opinion implicates the importance of physical, cognitive, affective and socio-occupational factors in the overall presentation of the patient which has prompted a call for a theoretical model shift within which to base the principles of physiotherapy [12]. The call for a shift to a patient-orientated biopsychosocial framework from a pathoanatomically-based therapist-orientated framework appears congruent to a patient-centred clinical method [13]. For example, the Quality Assurance Standards [14] refer to the need to care for the whole person in the context of their values and position in the wider community, with physiotherapists expected to make decisions collaboratively with patients and consider a range of therapeutic options for a particular health problem. The emphasis on holistic practice is positioned alongside the expectation that they will adopt a critical and evidence-based approach to care. How these two tenets of UK based health policy are resolved remains to be seen. Recent research, however, indicates a growing recognition by physiotherapists of the importance of integrating a ‘biopsychosocial’ approach in back pain management [10,11], and the research reported here explores how physiotherapists achieved this, particularly in relation to how they attempted to balance the mechanical and the psychosocial components of back pain care with the patient.

## Methods

### Context

The qualitative research reported in this paper was part of a larger study, the BeBack study (‘Beliefs about Back pain study’) aimed at understanding the attitudes, beliefs and behaviours of GPs and physiotherapists in the UK about managing patients with low back pain (LBP). The research comprised a national survey and an embedded qualitative interview study. The survey questionnaire included demographic and practice items, a measure of attitudes and beliefs about back pain, and items on intended clinical behaviour. Clinical practice behaviour was contextualised by providing a vignette describing a patient with uncomplicated non-specific low back pain, but who was not at work due to their back pain [15]. Respondents to the survey provided their recommendations regarding work, activity, bed rest and diagnostic investigations for the patient described in the vignette. The vignette was also used as part of the interview schedule to help contextualise the questions in the original qualitative interview study. Ethical approval was obtained from the West Midlands Multi-Centre Research Ethics Committee. Key themes from the primary analysis of the GP and physiotherapist interviews have been published elsewhere [15,16], however this paper reports findings that have not been previously published and report how physiotherapists navigate the competing pressures of evidence based practice and holistic care. Interviews were carried out between September 2005 and February 2006 lasting between 30 and 60 minutes. They were digitally recorded and fully transcribed. Following subsequent discussions with the research team, the decision was made to reanalyse the interviews using secondary analysis techniques (see below), in order to address a new research question concerning how therapists attempt to resolve the competing pressures to address both the mechanical and the psychosocial components of back pain care with the patient. The secondary analysis uncovered new insights not reported previously in the literature.

### Sampling and interviews

Semi-structured interviews with practicing physical therapists were conducted. Interviews were nested within a large national survey of the self-reported clinical management of healthcare professionals. In total 4,000 physiotherapists and GPs in the UK with recent experience of managing patients with LBP, were sampled randomly from national databases. From a total of 580 responding physiotherapists participating in the survey (response rate = 55%) 14 physiotherapists were purposefully sampled for the primary interviews according to their demographics (e.g. gender and age), time since qualification, clinical seniority or grade, and previous experience of managing LBP, as well as their treatment orientation and reported behaviour from the survey results. Purposive sampling was employed for maximum variance. From fourteen physiotherapists who were approached by an invitation letter and information sheet, 11 consented to an interview (by telephone), after which time no new insights emerged from the data. We also interviewed an additional physiotherapist, from a local physiotherapy clinic (providing informed consent) for a pilot interview prior to commencement of the interviews, and the data from the pilot interview were used as part of the secondary analysis presented in this paper, though not as part of the primary data analysis. The respondent characteristics are summarised in Table 1 below. The issues covered in the original interviews were: the types of patients seen, thoughts about LBP and any change in thinking over the course of their career, perceptions about how patients respond to LBP, treatment approaches and patients’ expectations.

**Table 1:** Characteristics of physiotherapists

**ID**	**Gender**	**Years since qualification**	**Work**	**Work context**	Grade*	**Agenda for change band**
6983	Female	22	Non NHS	Lone	N/a	N/a as non NHS
6920	Female	4	Non NHS	Multi-disciplinary	Staff Grade	N/a as non NHS
6900	Female	11	NHS	Multi-disciplinary	Senior II	Band not allocated when surveyed
5997	Female	33	NHS	Lone	Senior I	Band 7
5983	Female	10	Non NHS	Lone	N/a	N/a as non NHS
5960	Male	26	Both	Lone	Extended scope practitioner	Band 7
5499	Male	19	Both	Others	Extended scope practitioner	Band not allocated when surveyed
5393	Male	19	Non NHS	Others	Consultant	N/a as non NHS
5332	Male	16	Both	Others	Senior I	Band 7
5324	Male	12	Both	Lone	Senior I	Band not allocated when surveyed
5317	Male	8	NHS	Others	Senior II	Band not allocated when surveyed
B5544	Female	n/a	NHS	Others	n/a	n/a

*Staff grade is newly qualified, typically up to two years’ experience. Further grades are graded according to clinical, managerial and supervisory responsibilities ranging from Senior I to consultant therapist. Grades have subsequently been changed to bands within the Agenda for Change Framework (Agenda for Change Final Agreement. Department of Health; 2004).

### Analysis

A secondary analysis of the data was conducted by a researcher (TS) who was not involved in the original interviews, and had not seen the previous findings. This provided a degree of objectivity to the analysis. The coding and early findings were discussed at regular intervals with the other authors, to check the data for reliability and credibility. An inductive approach to the secondary analysis was adopted, where themes and interconnections between them were derived from the data [17]. Potential themes were explored through close reading of the interview data, and were subsequently discussed at meetings to establish their importance and relevance to the main focus of the secondary analysis. Secondary qualitative data analysis is increasingly adopted to shed new light on ‘primary’ data through a novel perspective or to deepen understanding of the phenomena under investigation [18]. The rationale behind qualitative secondary analysis is that primary findings can be viewed in a different way in light of new developments in the literature (both research and theory). This is not to disprove the previous findings but to offer new insights on the data. Original data analysis may have been conducted for different purposes, such as to provide a simple description of respondents’ views about managing patients with low back pain, whilst secondary analysis could be conducted using a different conceptual framework to highlight a particular ‘problem’ or question in the data that could be highlighted as having particular relevance to care delivery or patient outcomes. In our case, the frame that we used to reanalyse the data was the ‘biopsychosocial’ model. The transcripts from our interviews were re-analysed using a new coding frame that reflected the senior researcher’s (TS) new interpretation of the data, and were coded using QSR’s N-vivo system to develop a coding scheme, for later discussion with the research team. We looked afresh at the data specifically for the ways in which physiotherapists explored the emotional, cognitive, and social dimensions of pain in patients with LBP. This involved a combination of inductive and deductive reasoning. For example, we wanted to explore how physiotherapists managed patients’ emotional reactions to illness, such as fear or anxiety, as well as patients’ cognitive responses, including reasoning and understanding of their pain symptoms and how they were managed during routine physiotherapy encounters. We also sought evidence for ways that physiotherapists explored the impact of or the impact on, patients’ social context, such as financial pressures or support received to manage their pain symptoms (inductive). The published literature on the psychosocial model of care provided us with a coherent framework for analysing the data (deductive reasoning). Secondary coding and analysis was conducted by the researcher (TS) in the first instance, in isolation of the findings generated earlier during the primary analysis stage. Subsequently, the original transcripts were coded independently by two researchers and discussed at regular meetings (NF and BNO), in order to determine the extent to which we agreed to the interpretations of the interviews. Full agreement was reached on the findings reported here following discussion. We ensured that our main findings were robust through systematic comparison of the interview transcripts, which involved reading them in depth and comparing the dominant themes in each transcript. This enabled us to detect similarities and differences across all transcripts. The research team were confident that significant new insights were unlikely to emerge with additional data collection to aid the secondary analysis. We reached the decision that the primary data were sufficient to draw our conclusions.

A key theme arising from the secondary analysis was the distinction respondents made between mechanically focused and psychosocial care, which we contrasted across all interviews and were able to describe similarities and differences in relation to the distinctions they made. We also identified several sub-themes which overlap to varying degrees.

The stance we take here, by the nature of the methods employed in the study (therapists’ perceptions of practice) is that physiotherapists’ views are not necessarily a true reflection of their behaviour in clinical practice, though they do offer important clues about how clinicians understand their role. Here, we report re-interpreted interview material relating to the psychosocial and physical management of patients with LBP. The quotations were selected to illustrate the main themes and not to provide a representative selection of quotations across all respondents. The results are divided into two broad sections describing the perceived obstacles to recovery and physiotherapists’ responses to them.

## Results

### Theme 1: Perceived obstacles to recovery

a) The importance of psychosocial factors

LBP was thought to give rise to negative emotional responses in sufferers, such as panic, fear and anxiety. For this reason physiotherapists sought to ‘resolve’ some of these emotional reactions in patients. Although they considered such responses to be common amongst people consulting with LBP, they also thought that they should be properly addressed and patients offered reassurance about their symptoms.

B5332: Just through experience, you know, is that there are some joints that physios would call emotional joints… there’s a lot of panic with patients as well and you know and fear. They don’t know what’s going on. “Could it be serious? Could it not be?” and I think what they’re looking for is for you to say “look, this isn’t serious, it’s mechanical, we can help you.”

The management of physical symptoms was thought to be closely connected with patients’ lay beliefs about the causes and manifestations of LBP. While physiotherapists recognised the psychosocial aspects of the LBP experience and the associated link with these factors and chronicity, they claimed to lack sufficient training to effectively address these concerns with patients.

5960: But I’m not claiming that I can help all those patients. I mean there is a limitation to what I can achieve with regard to my, say, counselling skills and my skills of helping them with modifying their pain behaviour and helping them with their cognitive, you know, construct if you like, regarding low back pain. So, and in those cases if it’s outside my area of outside my boundaries if you like, I tend to involve other professionals as well.

This therapist recognised the importance of addressing patients’ psychosocial concerns. However, physiotherapists struggled to find strategies to integrate the clinical explanations within a broader biopsychosocial framework that made sense to patients.

b) Legitimacy of LBP

LBP is often associated with work absence. Thus it may be unsurprising that wider cultural connotations influenced the beliefs of our respondents towards some patients, and perhaps reinforced the view that they could not always be helped by physiotherapy management alone. Some physiotherapists suggested that they felt certain patients were financially dependent on having LBP. The following respondent alluded to the possibility that patients in receipt of financial support for their back pain or disability could be less likely to follow treatment advice from physiotherapists.

B5332: I suppose, I mean, if you really went down to it, you could talk about those people who are, or you know, poverty in patients, little money, sometimes, is quite, you know, they’re quite willing to be ill, if you understand me?

Interviewer: Yeah, yeah, and the reasons for that?

B5332: Maybe their own benefits, they will be earning more through that way rather than going back to work.

Interviewer: Right, right. So then that makes it, yeah, quite.

B5332: Yeah, but although I’m saying that, it’s very hard to prove anything. You only have your suspicions. There’s nothing you can actually, you can’t turn round and accuse patients of being like that.

These factors clearly impacted on the way physiotherapists viewed patients, and how far they were prepared to persist with those who perhaps were not making progress as expected or who were unwilling to follow clinical recommendations. Lack of progress was sometimes construed as refusal by the patient to follow the physiotherapist’s clinical recommendations. These responses may reflect a wider perception of LBP as attracting largely negative stereotypes of sufferers, and which potentially detract from a person-orientated approach to management. Such views could lead to the marginalisation of patients’ concerns, with greater clinical attention invested in those who appear to want to follow clinical advice. In such circumstances, physiotherapists may find it helpful to open up discussion with patients about these sensitive topics, in order to fully understand patients’ motivation to follow the clinical recommendations.

### Theme 2: Responding to psychosocial ‘obstacles’

a) Negotiating psychosocial problems

Physiotherapists found patients’ unrealistic expectations about the likely success of treatment difficult to manage during consultations. Although they recognised the importance of discussing psychosocial obstacles to recovery with patients, they also stressed that patients had a duty to follow the physiotherapy advice and acknowledge responsibility for their own LBP rehabilitation.

B5997: You certainly get a gut feel of the ones that you’re wasting your time on…They perhaps think they’re coming to me for a massage or something to be done to them to make them feel better, and I won’t go along that line. So they are difficult and there are times when I’ve had to say “well, look if you don’t want to follow what I’m saying I’m afraid I can’t help you.”

The need to identify key psychosocial obstacles to recovery was stressed by physiotherapists, though they expressed difficulty in fully dealing with and managing these obstacles, perhaps preferring to maintain a level of professional detachment. Some patients sought a quick resolution and did not recognise the non-physical ‘obstacles’ to their LBP recovery. Physiotherapists conceded that pursuing a full recovery from LBP could be futile in these cases.

B5499: It could be things the patient has been told about their problem that makes them feel it’s something more serious than it is and you can’t, no matter how much you explain that, you don’t actually get through to them. So that can mean that the treatment you think is going to work doesn’t.

It was thought that LBP can acquire central prominence in a sufferer’s life, and encouraging deeper discussion and providing a more in depth explanation about what is expected of patients during the consultation was considered important to redress the balance.

B5332: I suppose when I when I first qualified, patients became more dependent on me because of the way I treated them but now it’s trying to get them to say, “look,” well, yeah “you have to help yourself. I can give you the information and I can I can help, maybe, get rid of your acute period at this point, but you’re the one who has to help yourself, here.” You know, so its trying to just reinforce that bit, doing it in a nice way and you know, and being empathetic with them. They have got pain, but they have to realise that they have to then maybe do some pain management as well. That we might not be able to take the pain away fully but you need to get on top of it, break your kind of cycle here.

b) Addressing lay health beliefs

Illness beliefs can influence decisions to engage or abstain from health enhancing activities [19-21]. Health professionals may be aware of the clinical agendas but may be less familiar with the patient’s agenda. In the current study, this view led physiotherapists to try to ‘reposition’ patients’ health beliefs in line with current understanding of the pathoanatomy and mechanics of LBP.

B5544: If we say “you’ve got a degenerating spine” I think if people think that using it is going to degenerate faster, and unless you actually tell somebody that’s not the case then you can’t blame them for believing that…

This physiotherapist recognised the potentially negative impact of the language used during LBP consultations, and stressed the need to ensure patients were not misinterpreting the clinical message. Several physiotherapists claimed that many people still believe that exercise is ‘harmful’ for LBP, and the most helpful approach during physiotherapy management is one that reinforces the message that exercise and physical activity are actually central to recovery.

B5997: Well, she’s had this for four weeks. The benefit of having time off at this stage is minimal compared to maybe the first week of the back pain, and it would be more important to make sure that her work posture is correct, rather than just to avoid work. I think at this stage four weeks after the initial onset she needs to be mobile, not resting.

Quite to what extent physiotherapists relayed this message to patients during interactions is unclear as we did not conduct observations. Despite recognising the importance of the psychosocial dimension of LBP, physiotherapists questioned the appropriateness of engaging patients in such discussions and anticipated a certain level of resistance. The following quote provides an example of how one physiotherapist did engage with a patient’s ‘unhelpful’ reaction to her LBP, (i.e. anxiety and fear of movement) which is presented as something to be corrected through specific advice.

B5324: With this lady there’s anxiety problems there, she’s demonstrating past coping mechanisms, she’s got a fear of flexion. These are all things that you can address by explaining to her that that’s exactly the wrong way of going about dealing with back-pain, and then its whether they then actually buy into that and accept that and start doing it.

The emphasis was on helping the patient derive a realistic understanding of their back problem to facilitate recovery. This was achieved through presenting ‘hard’ ‘evidence’ such as MRI results to objectively demonstrate the presence or absence of serious spinal pathology.

B5393: With the chronic ones who tend to be imaged to death, so you can be quite sure that we know there’s nothing wrong with you because, you know, you’ve been scanned; you know, you’ve had an MRI, you’ve had a CT (scan), whatever. You know, as far as medicine’s concerned you’ve had blood tests; there’s nothing wrong with you, so, we know what it is and we could get on with it and if you buy into this we’ll get there.

Psychosocial issues were perceived central alongside biomedical factors, to managing back pain by our participants. However, the aim was to discourage the behaviours or attitudes of patients that could prevent recovery, through promoting health enhancing practices such as losing weight.

B5332: I think you’ve just got to ‘plug away’ at them; just got to say, “well” you know “you might think you’re right, but evidence is suggesting that if you lose some weight then we can make a difference in your back.”

Physiotherapists encouraged people to live with their pain, rather than attempt to understand the underpinning reasons for their ‘negative’ beliefs. Others encouraged patients to describe their concerns and fears in the hope that they could be redressed through discussion. Again the emphasis seemed to be on putting ‘negative’ beliefs to rest through reassurance. However, reassurance alone may be insufficient to eradicate the intractable psychosocial problems.

B5544: I probably won’t treat you today. I may not even going to look at you today. “I’m going to listen to you” and by letting them talk and letting them tell me absolutely everything, and keeping it as unstructured as possible, hopefully, as I say, we’ll find these little bits of things that have either been missed or have never ever come out in the open, you know and it can be the smallest, smallest little fear that’s niggling away in somebody’s head and you can have dramatic effects just by *putting that to rest*.

c) Setting boundaries

Discussing and negotiating lay health beliefs with patients was one strategy used by physiotherapists to aid recovery. However, such an approach was not always successful because patients either did not appreciate the advice or resorted to old ways of thinking about their back pain and its management. Consequently, a further strategy was for physiotherapists to place boundaries around the management of psychosocial problems, in the realisation that the negotiated approach was not always effective.

B5324: If there’s a relationship issue and things like that, that’s stuff that I won’t add, won’t necessarily address, because I don’t think that’s my area. I mean, I’m not going to start saying to patients, you know, how is your relationship with your husband at the minute, because I don’t think that’s the, it’s certainly relevant, but what am I going to do about it, if you know what I mean? If they start ‘bringing up’ those sorts of issues?

Physiotherapists were reluctant to engage in discussion regarding patients’ personal lives in the absence of the necessary skills with which to offer appropriate advice. Involving other professionals with greater knowledge of psychological problems was one solution.

B5499: looking at why the patient’s anxious, why they’re not doing things. Sleep patterns, all those kind of things that you look at but I think its limited as to how far you go with that. The patient’s presenting with a very chronic pain pattern and I think if that’s the case, they’re better treated with a multi-professional input because I think the outcomes then are much better than just seeing the patients as a sole professional.

Others claimed that in certain circumstances, for example where people received financial benefits as a consequence of their disability, the challenge of assisting patient recovery from their back pain was thought to be too great. This was because patient’s wanted to maintain a ‘back pain identity’ with which to justify the presence of incapacity, and hence demonstrate inability to resume normal activities.

B5544: I’ve got one gentleman, at the minute, all he wants is his benefits and you know, realistically you can find absolutely nothing wrong from an assessment but all he wants “I want my…” you know “I want my benefits, I can’t work because you know I had this injury 15 years ago.”. OK yeah but that was 15 years ago, but he’s now got a full range movement of his spine and he could, quite happily work, but he doesn’t want to. He wants to stay quite disabled and, you know we’re on to a non-winner with that one…

Others claimed that physiotherapists perpetuated a ‘back pain identity’ in patients by reinforcing their physical ‘incapacity’ rather than their ability to adapt to life with pain.

B5317: We keep asking them, “well, how long can you walk for?” or “how many of these can you do before your pain comes on?” and then they go and analyse that and they’re actually looking for the pain constantly. So, I think, sometimes we in breed that in people.

The implication was that physiotherapy practice should be an approach that does not focus on the pain response. In this case, boundary setting was a strategy with a dual purpose for physiotherapists; to help define their scope of practice more explicitly for greater clarity about their clinical role, and to aid patient recovery through a focus on patient self-care by encouraging them to focus less on pain symptoms and limitations and more on function and activities they can achieve. In other words, a greater focus on what they can do rather than what they cannot do.

## Discussion

The management of LBP using a biopsychosocial approach has been endorsed by institutions such as the National Institute for Health and Clinical Excellence [1], and steps have been taken to improve understanding of what such an approach might look like [22,23]. Although our physiotherapists recognised the importance of patients’ social and psychological contexts, they were also fully aware of the challenge that such matters presented. Many referred to the difficulty of dealing with patients’ lay health beliefs. Psychosocial ‘obstacles’ were viewed as potentially inhibiting patients to follow therapeutic advice. Despite these difficulties physiotherapists acknowledged the importance of engaging in negotiations with patients about the full range of biopsychosocial obstacles to recovery, though they did not feel they possessed adequate skills or training to deal effectively with psychosocial obstacles specifically. This finding has been reported previously [24].

The dominant characteristic of the physiotherapy paradigm which is focused on biomechanically oriented treatment approaches shaped therapists’ thinking about psychosocial issues. Addressing behaviour change rather than exploring lay health beliefs is wholly consistent with this paradigmatic approach [2]. Facilitating behaviour change through advice and education has been a major challenge for clinicians [15]. There seems to be a disconnection between what physiotherapists perceive to be clinically helpful health advice and patients’ own beliefs towards managing back pain. Patient engagement with therapists’ advice is central to maintaining a productive working relationship [15]. Discord between therapist and patient beliefs, however, could affect patient recovery. A strong patient partnership was therefore considered to be vitally important, and the threat of patient ‘conflict’ may have prevented therapists from recommending certain types of advice to patients to avoid undermining the therapeutic relationship. Further research on the strategies to be used by physiotherapists to manage the physical symptoms whilst utilising a psychosocial approach to care is now needed.

Physiotherapists also set boundaries for their clinical role in helping patients’ LBP, which allowed them to practice within their areas of confidence, focusing on physical issues (largely mechanical in nature) in favour of psychosocial obstacles to recovery. Though both components were perceived as important to patient care, during routine clinical practice they were largely considered as distinct issues. The unpredictability of the course of LBP and response to treatment exacerbated the feeling amongst physiotherapists that it could pose an intractable problem. This has been reported previously [24]. Jeffrey and Foster (2012) concluded (using data from the same dataset reported here) that LBP was seen by physiotherapists largely as a structural or mechanical problem. The findings reported in this article are an extension of this stance, and show that physiotherapists also attached importance to the need to identify and address patients’ psychosocial problems, though with variable success. We interpret the findings reported here and those of Jeffrey and Foster (2012) as supporting the view that physiotherapists recognise the need to address the psychosocial needs of patients, but often feel more competent and confident to prioritise presenting physical problems. We also recognise that LBP is a multidimensional issue and physiotherapists often attempt to integrate the physical and psychosocial problems within a single coherent package of care, though the physical problem may often take priority. Third, the challenge of ‘legitimacy’ also affected physiotherapists’ approach to LBP, sometimes leading to negative perceptions about the ‘types’ of patient suffering with back problems, and their potential hidden agendas. The conflict between the traditional physiotherapy paradigm with its emphasis on back pain as a ‘mechanical’ problem, and engaging with patients’ additional psychosocial problems, referred to by many of our respondents as ‘obstacles to recovery’, seemed to be left unresolved. The interaction of social and psychological issues with patients’ back pain symptoms, was clearly viewed by physiotherapists as increasing the complexity of the patient case, but they felt they lacked the necessary skills with which to satisfactorily identify or address them.

### Practice implications

The findings confirm the recognition by physiotherapists of the need to identify and address the social and psychological obstacles to recovery that patients with low back pain experience. They struggled, however, to find ways to understand and address these psychosocial factors, and often claimed that these problems fell outside of their immediate scope of practice. It is important to recognise the distinction between psychosocial factors or obstacles affecting patients’ ability to manage their back pain, and their *health beliefs*, which may be more difficult to moderate. Consequently, there is a need for physiotherapists to both recognise patients’ health beliefs, and respond with appropriate clinical advice. Providing advice without fully understanding patients’ beliefs could lead to poor ‘concordance’ or recovery from back pain. This is because patients may respond negatively to clinical recommendations that do not sufficiently connect with their experiences of living with back pain. The STarT Back trial showed benefits from stratified care in outcomes such as back disability/function as well as key psychological obstacles to recovery like fear avoidance [25]. Health beliefs, such as a fear of movement for example, may benefit from acknowledgement (‘validation’) by the physiotherapist as well as a therapeutic resolution. Thus, good communication is important given that physiotherapists need to develop effective ways of eliciting these obstacles to recovery such as fear avoidance beliefs. Consequently, a change in attitudes regarding the health beliefs of patients and their psychosocial concerns may shift the clinical focus from only mechanical management to identifying appropriate communication approaches; based on the view that communication is an integral component of physiotherapy practice [See Foster and Delitto 2011 for examples of physiotherapists’ use of the biopsychosocial model]. The interviews also suggested that LBP sometimes attracts negative stereotypes of patients, which could detract from a person-orientated clinical approach to communication, and perhaps lead to greater clinical attention invested in those who appear to want to follow the clinical advice. The findings therefore point to a need to develop approaches to help physiotherapists feel more confident and competent to identify key psychosocial obstacles to recovery, acknowledge and legitimise patients’ concerns and structure their communication with patients in ways that more closely acknowledge patients’ beliefs. Physiotherapists are ideally placed to manage these different dimensions of back pain work, partly because of the time they can dedicate to communicating with patients compared with GPs, helping them to acquire a holistic view of the patient and their LBP.

### Recommendations for future research

This secondary analysis was based on interviews carried out with a sample of physiotherapists. The central issue is the integration of the best of what we know about mechanical components of back pain with the best of what we know about the key psychosocial factors. Studies show that factors such as satisfaction with care often improve with a psychosocial focus in the consultation [26,27]. Moreover, there is a need to conduct direct observations of physiotherapist-patient consultations to compare clinicians’ and patients’ accounts with actual practice.

### Strengths and limitations

The physiotherapists in our study were sampled purposively from respondents to a national survey ensuring that we captured a breadth of characteristics [17]. The findings report on an area of physiotherapy practice that has received limited research attention so far, and which offers suggestions of how physiotherapy might be delivered as part of a broad biopsychosocial model of care. Secondary analysis provides the opportunity to re-examine qualitative data in light of new ideas, recent research evidence, and developments in policy and clinical practice. However, the analytical process can also be demanding if conducted by an independent researcher, who was not involved in the original data collection and analysis. Conversely, a fresh pair of eyes and experience helped to uncover new insights in our data. Although the decision to reanalyse the data had come from insights developed following initial data collection, caution is required when interpreting some of the findings. For instance, the views expressed by our physiotherapists may not necessarily reflect current practice. Though, it is unlikely that current practice would have changed radically since the interviews were conducted. The NICE back pain guidelines cited in the paper were published in 2009 calling for greater use of the biopsychosocial model, which is over three years since the initial interviews were conducted. Therefore we cannot expect our interviewees to reflect this change in their responses. However, it is reasonable to conclude that many physiotherapists had begun to recognise the importance of the biopsychosocial model in back pain care prior to the NICE guideline, with research revealing the possible connection between patients’ psychosocial factors and clinical outcomes [28-32]. Analytical rigour was strengthened through a process of regular cross validation, where emergent themes from the secondary analysis were coded independently by the research team and discussed at regular meetings. This helped to strengthen the authenticity and credibility of the thematic framework as well as to clarify insights where there were disagreements or alternative explanations. Secondary analysis provides the opportunity to re-examine qualitative data with a ‘fresh pair of eyes’ ensuring new insights and improved objectivity. The process also enabled us to build on previous findings. We believe that this approach has greatly strengthened the level of analytical rigour and explanatory power of our data.

## Conclusions

The findings show that physiotherapists recognised the centrality of patients’ psychosocial context and the ‘social’ mediators of back pain, such as work absence, which played a key part in affecting patient recovery. The implications of these findings are that in order to achieve concordance with patients physiotherapists will need to use best practice recommendations in ways that take account of patients’ pain experiences [30]. In other words a balance between patients’ psychosocial issues and biomedical approaches to managing patients’ pain problems are needed, though as yet physiotherapists may struggle to adopt strategies to identify and manage both the biomedical and psychosocial aspects of non-specific back pain that patients present with. If formal evidence-based practice considers alignment with patients’ psychosocial and biomedical concerns as a central aim, then physiotherapists may need to develop strategies for eliciting and addressing them as part of an integrated package of care [31,32].

## Competing interests

The authors declare that they have no competing interests.

## Authors’ contributions

TS conducted the initial steps/components of the secondary analysis and drafted the manuscript. NEF and BNO contributed to the analysis and all authors contributed to interpretation. All authors read, contributed to, and approved the final manuscript.

## Pre-publication history

The pre-publication history for this paper can be accessed here:

http://www.biomedcentral.com/1471-2474/14/65/prepub
